# Gel Properties and Structural Characteristics of Composite Gels of Soy Protein Isolate and Silver Carp Protein

**DOI:** 10.3390/gels9050420

**Published:** 2023-05-17

**Authors:** Li Zheng, Joe M. Regenstein, Linyi Zhou, Sayed Mohamed Mokhtar, Zhongjiang Wang

**Affiliations:** 1 College of Food Science, Northeast Agricultural University, Harbin 150030, China; wzjname@neau.edu.cn; 2 Heilongjiang Beidahuang Green Health Food Co., Ltd., Kiamusze 154007, China; 3 Department of Food Science, Cornell University, Ithaca, NY 14853, USA; jmr9@cornell.edu; 4 School of Food and Health, Beijing Technology and Business University, Beijing 100048, China; 5 Department of Food Technology, Faculty of Agriculture, Suez Canal University, Ismailia 41522, Egypt; smmokhtar@yahoo.com

**Keywords:** soy protein isolate, silver carp protein, papain, glutamine transaminase, *Glycine max*, *Hypophthalmichthys molitrix*

## Abstract

Problems with silver carp protein (SCP) include a strong fishy odor, low gel strength of SCP surimi, and susceptibility to gel degradation. The objective of this study was to improve the gel quality of SCP. The effects of the addition of native soy protein isolate (SPI) and SPI subjected to papain-restricted hydrolysis on the gel characteristics and structural features of SCP were studied. The β-sheet structures in SPI increased after papain treatment. SPI treated with papain was crosslinked with SCP using glutamine transaminase (TG) to form a composite gel. Compared with the control, the addition of modified SPI increased the hardness, springiness, chewiness, cohesiveness, and water-holding capacity (WHC) of the protein gel (*p* < 0.05). In particular, the effects were most significant when the degree of SPI hydrolysis (DH) was 0.5% (i.e., gel sample M-2). The molecular force results demonstrated that hydrogen bonding, disulfide bonding, and hydrophobic association are important molecular forces in gel formation. The addition of the modified SPI increases the number of hydrogen bonds and the disulfide bonds. Scanning electron microscopy (SEM) analysis showed that the papain modifications allowed the formation of a composite gel with a complex, continuous, and uniform gel structure. However, the control of the DH is important as additional enzymatic hydrolysis of SPI decreased TG crosslinking. Overall, modified SPI has the potential to improve SCP gel texture and WHC.

## 1. Introduction

The world’s population is expected to reach 11.2 billion by 2100. This will lead to increased meat consumption, especially in developing and emerging countries [1]. The growing demand for meat will continue to drive the development of the livestock industry in the future. Industrial livestock production has been suggested to be one of the main causes of climate change and loss of biodiversity [2]. Many studies have been looking at how to balance the relationship between food and the environment. The focus is mainly on two aspects. One is active research into the source and function of plant proteins that can partially replace animal proteins, such as soy and rice proteins. The other is the exploitation of the existing wasted but valuable animal proteins, such as silver carp protein (SCP). China is the world’s largest producer of freshwater fish [3], and silver carp (*Hypophthalmichthys molitrix*) is one of the “four major freshwater fish in China” [4]. The silver carp has the largest biomass and its price is low, so the commercial value of fresh silver carp is low [5,6]. The use of silver carp as a raw material for the production of freshwater surimi is growing [4]. The development of protein feeds using silver carp as a resource not only increases the value of silver carp but can also promote the rapid development of freshwater fish farming and its processing [6].

However, SCP has issues, such as a strong fishy smell and a low gel strength of SCP surimi, which is prone to gel degradation [7,8,9]. While challenging, it also brings more opportunities for researchers to develop systems to improve SCP gel quality. Flesh quality and flavor characteristics are important for the manufacture of fish gel products. The current approaches to improve fish quality have not increased market demand. Therefore, research into improving the quality as well as delaying gel degradation during the formation of SCP gels could be beneficial. Several factors may affect the composite gel quality. These can be divided into intrinsic and extrinsic factors. The quality of fish gel products could vary from one species to another, or even within one species due to different intrinsic factors. Thus, previous work with other species and with the same species still needs to be confirmed and expanded. Based on the above analysis, SCP has been studied further. To realize the potential of silver carp meat as a low-fat and high-protein food source for high-quality fish products and to fully exploit its value [10], new methods need to be developed. This study’s goal is to improve gel quality.

Taking into account sustainability and nutrition suggests that adding a plant-based protein might have multiple benefits. Among the many plant proteins, soy protein isolate (SPI) has many advantages, such as providing a good amino acid balance and low price [11,12,13]. SPI is often used in sausage, ham, and tofu products [14,15]. Native SPI (N-SPI) may not be a good additive for meat gels due to its weak binding ability with other proteins and its potential to sometimes weaken these gels’ properties [16]. It has been determined that meat emulsions could be significantly improved both for texture and nutrition using enzyme-modified N-SPI [17,18,19,20]. Many factors affect enzyme modification such as protein source, enzyme type, and hydrolysis conditions [18]. Papain-modified N-SPI has good gel properties [21,22] suggesting its use for composite gels with SCP to have the best quality gel products, with maximum yield.

The effects of different degrees of hydrolysis (DH) of the papain-restricted hydrolysis of SPI on the gel properties and the structure of the SPI and SCP composite gels were studied. Composite gels were studied for their textural properties, structural characteristics, color, WHC, intermolecular forces in the gel, microstructure, and sensory properties using Fourier-transform infrared Spectroscopy (FTIR), texture profile analysis (TPA), and SEM. The results will provide theoretical guidance for the application of SCP composite gels in food systems, and effectively improve the functional properties of food.

## 2. Results and Discussion

### 2.1. FTIR Spectroscopy

The main secondary structure of SPI is β-sheets, which is consistent with Li et al. [21] and Zhao et al. [23]. As shown in Table 1, after papain hydrolysis, the contents of α-helices and β-sheets increased from 26.1 and 31 to 28.4 and 36%, respectively, while the contents of β-turns and random coil decreased from 25.4 and 18 to 22 and 15.2%, respectively. When the DH was 0.5%, the β-sheets increased to 36%. Wang et al. [24] found that after the hydrolysis of wheat protein using an alkaline protease, the contents of α-helices and β-turns decreased, while the contents of β-sheets and random coil increased. This may be due to the difference in protein secondary structure after protease action with different proteins. After enzymatic hydrolysis in this study, after adding TG, the increase in β-sheet structure may have been due to the hydrophobic groups exposed after hydrolysis crosslinking into a denser gel network structure. This is consistent with the measured gel texture changes. Therefore, it can be concluded that limited papain modification can change the secondary structure of proteins and promote TG crosslinked proteins into forming a dense gel network structure.

**Figure 1:**
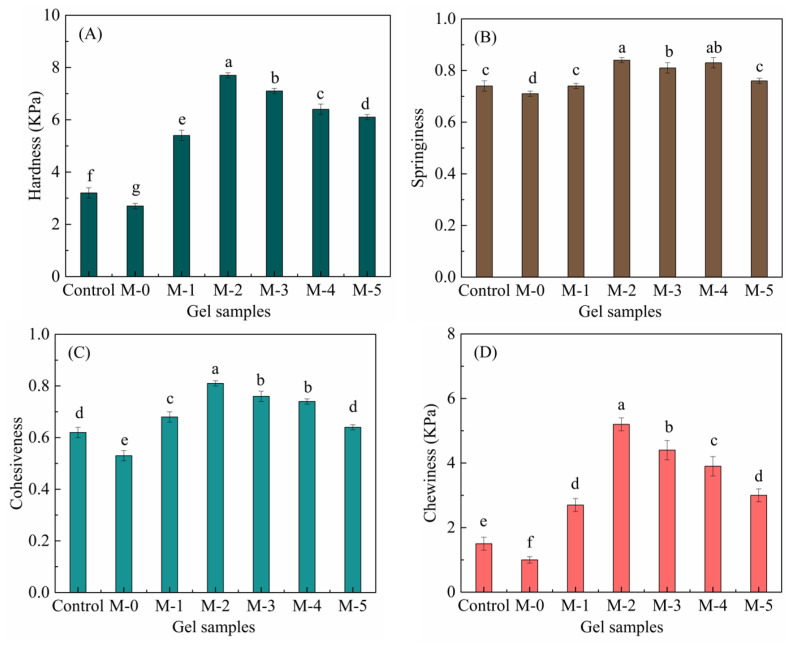
(**A**) The hardness of cold-induced gels formed with SCP alone, with SCP, and N-SPI, and modified SCP with SPI modified with papain at different DH (0.1, 0.5, 1.0, 1.5, and 2.0); (**B**) the springiness of cold-induced gels; (**C**) the cohesiveness of cold-induced gels; and (**D**) the chewiness of cold-induced gels; different lowercase letters indicate significant differences *p* < 0.05.

Previous studies [25,26] have shown that the β-sheet content of protein secondary structures has a significantly positive correlation with the hardness of different gel samples. In this study, composite gel M-2 has the highest β-sheet content and gel hardness (see Figure 1). Sow et al. [27] also pointed out that the increased content of random coil structures in gels may be related to poor gel texture characteristics (especially gel hardness). SCP gels and composite gels M-0 and M-1 had a higher relative content of random coils, and composite gel M-2 had the lowest relative content of random coils. The results of the gel texture measurements showed that the M-0 gel had the lowest hardness and the M-2 gel had the highest hardness. These results confirmed, once again, that N-SPI has a certain negative effect on improving the quality of SCP gels, and a modification technology is necessary. Comparing the secondary structure content to gel properties showed that the β-sheet content of the gels was positively correlated with the hardness of different gel samples (r = 0.911, *p* < 0.004), and the random coil content of the gel was negatively correlated with the hardness of different gel samples (r = −0.850, *p* < 0.015). Thus, the relationship between changes in protein secondary structure content and the texture properties may help to regulate composite gel hardness.

### 2.2. Analysis of the Textural Properties of the Gel Samples

Texture is an important quality of food gels and is related to the sensory quality of gel products. After partial hydrolysis of SPI with papain, the textures of the composite gels formed using TG crosslinking of SPI and SCP are shown in Figure 1. Hardness, springiness, cohesiveness, and chewiness were measured. The TPA method is often used for the measurement of gel texture due to the ease of obtaining multiple parameters, which reflect a range of food textural characteristics [28]. Hardness is the maximum peak load [29,30].

There were significant differences (*p* < 0.01) in the hardness values of the seven composite gel samples. The hardness of composite gels formed using modified SPI and SCP was significantly higher than that of the control and M-0 group. When the DH of SPI was 0.5%, the maximum hardness of the composite gel was 7.7 kPa. However, with increased DH, the hardness of the composite gels decreased. The study showed that enzymatic hydrolysis could change protein structure including exposing hydrophobic groups in protein molecules [21,31]. Papain’s specific lysine cleavage exposes more lysine residues, which facilitates the formation of a dense gel network through TG crosslinking. The uniform and tight microstructure can bind the water in the gel system, thus increasing the gel hardness and WHC. Similarly, Wu et al. [32] also reported that the uniform and dense microstructure of protein gels corresponded to higher gel hardness and WHC. In this study, when the DH of SPI was >0.5%, the hardness of the composite gels began to decline, possibly because the protein was over-hydrolyzed into smaller peptides, which were not able to crosslink with TG.

The texture properties of gels are mainly affected by the structure of the protein network, which may reflect differences in the secondary structure of proteins. There was a high correlation between gel hardness and β-sheet structure (r = 0.911, *p* < 0.004). The β-sheet structure was the main ordered structure of SPI. The greater the number of β-sheets, the denser the network structure of the protein gel and the higher the gel hardness [33]. The M-2 gel sample with the highest hardness has the highest amount of β-sheet structure, and the control group with the lowest hardness has the lowest number of β-sheets.

Other texture properties such as the chewiness and springiness of the gel samples also showed a similar trend. The M-2 gel sample had higher springiness and hardness, indicating a higher resistance to deformation and a better recovery of the shape. The M-0 gel samples had the lowest springiness and hardness, indicating poor deformation resistance and a lower ability to recover shape. Cohesiveness is a measure of how well a gel sample will withstand a second deformation relative to its resistance to the first deformation. Gel products with strong cohesiveness will be more able to withstand the pressure caused by manufacturing, packaging, and transportation, and, they will, therefore, better retain their characteristics to the end of manufacture. Chewiness reflects the effort required to masticate a solid sample ready for swallowing. More chewing energy is required in the mouth when the springiness and hardness values of the gel sample are high. In this study, the chewiness values of gel samples varied over a wide range. The chewiness of gel sample M-2 was the highest. In general, the higher the chewiness levels, the better the quality of the gel and the better it meets the expectations of the consumer [34].

SPI (DH = 0.5%), following papain hydrolysis and SCP that were crosslinked using TG, had stronger gels, with the springiness, hardness, chewiness, and cohesiveness of the cold-induced gels being greater than those of the cold-induced gels made at other DH. Furthermore, the results from the trained sensory panel showed that the composite gel M-2 had the highest scores for apparent state, taste, mouthfeel, and acceptability. The textural properties of modified composite gels were better than the control and unmodified composite gel groups.

### 2.3. Analysis of the WHC of the Gel Samples

**Figure 2:**
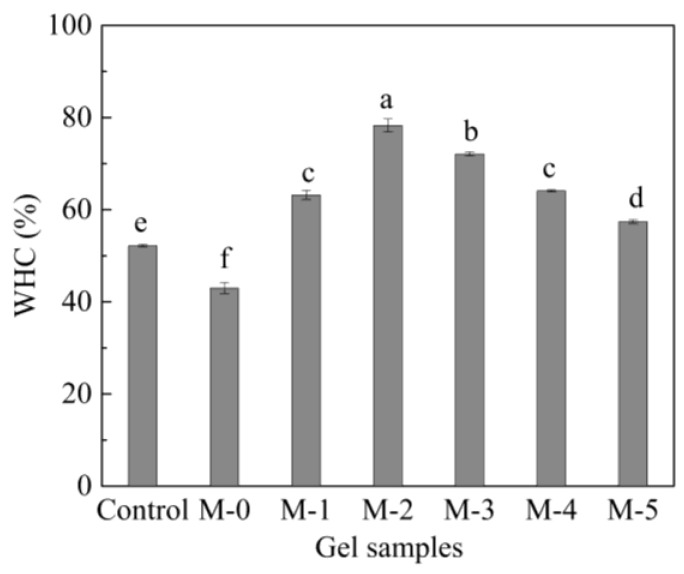
The effect of SPI modified with papain at different DH (0.1, 0.5, 1.0, 1.5, and 2.0%) combined with SCP on the WHC of compound gels. Different lowercase letters indicate significant differences *p* < 0.05.

The WHC of food gel products is an important functional property, which reflects the water-binding ability of proteins [35]. The WHC of different composite gels is shown in Figure 2. The WHC of the gels in the control group was 52.2%, and the WHC of the composite protein gels formed after the mixing of N-SPI and SCP was slightly reduced. When DH = 0.5%, the WHC of composite gels had its maximum value. The WHC of the compound gels decreased with increased DH. However, the WHC of all modified composite gels was higher than that of the control group, possibly due to water binding to the newly exposed lysines. The crosslinked TG cold gel retained more water. Kao et al. [36] also pointed out that the dense uniform microstructure of protein gels corresponded to higher gel strengths and WHC. In this study, the WHC of the composite gels began to decline when the DH was >0.5%, probably because of the smaller peptides.

### 2.4. Analysis of the Whiteness of the Gel Samples

**Figure 3:**
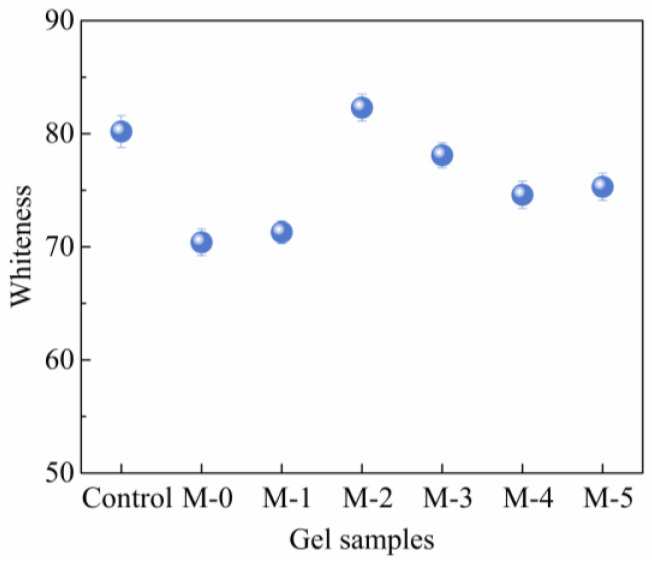
The effect of the SPI modified with papain at different DH (0.1, 0.5, 1.0, 1.5, and 2.0%) combined with SCP on the whiteness of compound gels.

**Figure 4:**
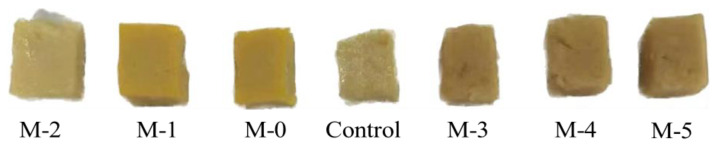
Images of the compound gels.

Assuming consistent product quality and product appearance can greatly influence the consumer’s willingness to buy [37]. The appearance of the protein gel can be an indicator of network formation. Gels formed by aggregating particles become opaque because the particles are larger and cluster more randomly, while gels formed by ordered structures (linear aggregation) are translucent. The whiteness of the opaque composite gel is shown in Figure 3 and the appearance is shown in Figure 4. After mixing N-SPI with SCP, the whiteness of the complex gel significantly decreased (*p* < 0.05), which may be because SPI itself was light yellow, and the whiteness of a complex gel decreased after mixing with SCP.

The decrease in whiteness value may also be related to the change in pigment protein. Niu et al. [16] and Saeed et al. [38] indicated that the decrease in whiteness might be caused by changes in pigment proteins, especially muscle protein oxidizing pigments. The whiteness of the modified compound gels was higher than that of the unmodified compound gels. In addition, with increasing DH, the gel whiteness showed a similar trend to WHC, which may be due to a higher water content brightening the flesh color [39].

### 2.5. Analysis of the Chemical Interaction Forces of the Gel Samples

**Figure 5:**
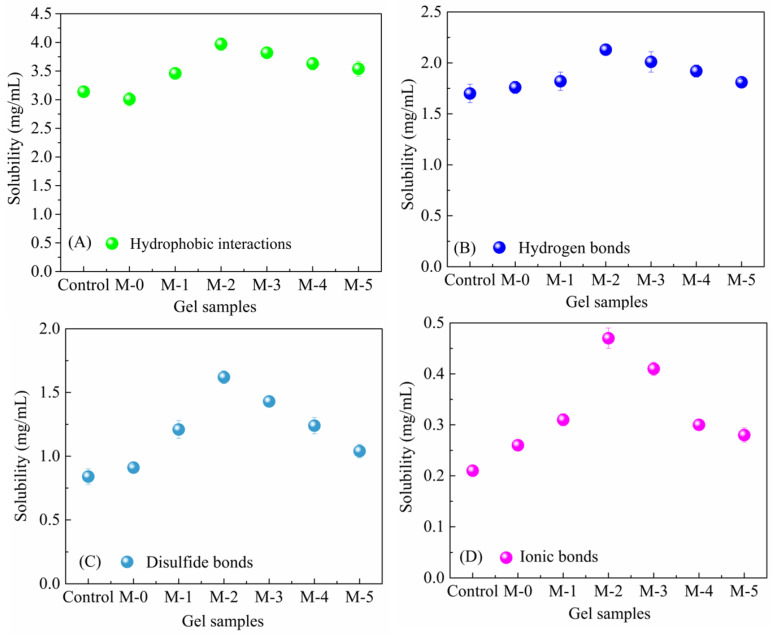
(**A**) The hydrophobic interactions of cold-induced gels formed with SCP alone, with SCP and N-SPI, and modified SCP with SPI modified with papain at different DH (0.1, 0.5, 1.0, 1.5, and 2.0); (**B**) the hydrogen bonds of cold-induced gels; (**C**) the disulfide bonds of cold-induced gels; and (**D**) the ionic bonds of cold-induced gels.

Typical protein gelation is mainly caused by the crosslinking of polypeptide chains that form a three-dimensional network stabilized by various molecular forces [40]. Niu et al. [16] pointed out that hydrogen bonding, disulfide bonds, and the hydrophobic association had important roles in protein crosslinking of a gel system of pork myofibrillar protein and modified SPI. The solubility of the control and composite gels in four different solvents was determined to obtain information about the types of bonding between protein molecules. Ionic bonds, disulfide bonds, hydrogen bonds, and hydrophobic interactions can be inferred. As shown in Figure 5, the composite gels formed by crosslinking SCP using TG after using papain-restricted hydrolysis of SPI gave values of hydrogen bonds, hydrophobic interactions, and disulfide bonds that increased while the ionic bonds decreased. This indicated that hydrogen bonds, hydrophobic bonds, and disulfide bonds may be more important than ionic bonds in the maintenance of the composite gel conformation stability, which is consistent with the conclusion reached by Wang et al. [41], who showed that the interaction forces that maintain the gel conformation are mainly hydrophobic interactions and disulfide bond interactions.

Regardless of the structural changes in SPI, hydrogen bonding is the fundamental force in mixed protein gels [16]. The content of all hydrogen bonds in the composite gels was higher than that in the control group, which confirmed the involvement of hydrogen bonds in the gel network. When papain-hydrolyzed SPI had a DH of 0.5, all of the bonds of the M-2 composite gel had the highest values. This may be due to the partial denaturation of protein molecules caused by enzymatic hydrolysis modification, resulting in protein decompression, the exposure of hydrophobic residues and internal sulfhydryl groups, the enhanced interaction between molecules, the increased protein hydrophobicity, and the formation of disulfide bonds [42]. In addition, the content of all disulfide bonds in the modified composite gels was higher than that in the control group. This showed that the restriction enzyme hydrolysis-unfolding-TG crosslinking–refolding procedure can enhance the modification effect. However, the modified composite gel (M-SPI/SCP) had more hydrophobic bonds than the unmodified composite gel (N-SPI/SCP), indicating that the hydrophobic residues in the modified composite gel have a positive role. Visessanguan, Ogawa, Nakai, and An [43] found that the exposed hydrophobic structural domain is a prerequisite for the formation of myosin aggregates. Therefore, based on the above analysis, it was concluded that hydrogen bonding, disulfide bonding, and hydrophobic association are the important molecular forces for the formation of composite gels. The addition of enzyme-restricted SPI enhanced the hydrogen and disulfide bonds of SCP.

### 2.6. Analysis of the Microstructures of the Gel Samples

**Figure 6:**
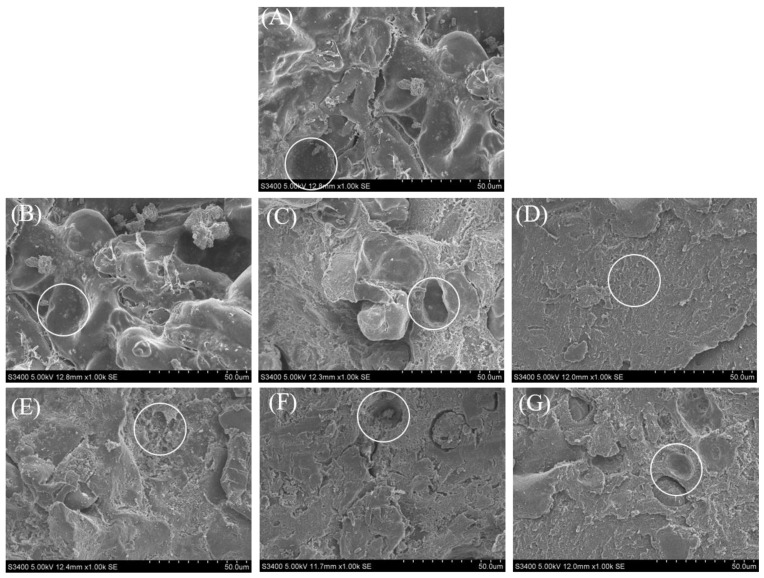
Scanning electron microscope images of composite gel samples with SPI at different DH: (**A**) Control SCP gels; (**B**) SCP gels with N-SPI; (**C**) SCP gels at a DH of 0.1% SPI; (**D**) SCP gels with a DH of 0.5% SPI; (**E**) SCP gels with a DH of 1% SPI; (**F**) SCP gels with a DH of 1.5% SPI; and (**G**) SCP gels with a DH of 2% SPI.

The three-dimensional network structure of the gel samples could be visually observed using SEM. Figure 6 shows the microstructure of composite gels formed with TG-crosslinked SCP and hydrolyzed SPI. The pores in the control group and the unmodified compound gel groups (Figure 6A,B) were larger than those in the modified compound gel groups (Figure 6C–G). The modified composite gel network was compact, especially when the DH was 0.5% (Figure 6D). As the DH increased, the network structure of the gels became larger, possibly because the disulfide bonds of the SPI were destroyed by the papain, and the structure was opened, exposing greater free SH and hydrophobic groups [27], thus promoting TG crosslinking into a dense gel network. However, with the increased DH, the formation of a dense gel network structure may no longer be possible. The smaller peptides might also lead to interactions between exposed active groups leading to re-aggregation, an increase in particle size, and a decrease in SPI solubility, thus leading to an uneven and rough surface. The microstructure of a gel system reflects the physical properties of the gel structure. Puppo and Anon [44] pointed out that protein gels with uniform microstructures and dense structures had higher WHCs than those with loose microstructures and rough structures.

### 2.7. Analysis of the Sensory Evaluations of the Gel Samples

Sensory evaluation using human evaluators can be used to measure many aspects of a product [45,46]. It can be combined with instrumental tests to give a more comprehensive evaluation of the tested products. However, it is also important to recognize that testers may have very different responses to a product that reflect cultural, nationality, and individual variations [47]. Panelist training leads to a more consistent response at the expense of consumer understanding to achieve greater objectivity in sensory evaluation.

Based on the rating scale in Table 2, the expert evaluators were asked to give a score between 1 (imperceptible) and 10 (very strong) for each evaluated characteristic. The scores are shown in Table 3. Compared with the control group gels, when the DH was 0.5%, the elasticity, mouthfeel, and taste of composite gels had the highest intensities (*p* < 0.05), which is probably related to the increase in the springiness and hardness of the composite gels. The apparent overall score of the modified composite gels was higher than those of the control group. The optimized hydrolysis treatment improved the gel microstructure and resulted in more uniform gel samples with smaller voids, according to Li et al. [21]. In addition, the whiteness of the modified composite gels was higher than those of the unmodified composite gels, which was consistent with the instrumental data for whiteness. For the overall acceptability, although no bitter peptides were produced in control gels and M-0 gels, the low scores were due to the taste of the samples. The overall acceptability scores for the M-1, M-2, and M-3 gel samples were high, indicating that the enzymes did not show significant bitterness when the DH was <1.0%. However, the M-4 and M-5 gel samples had reduced overall acceptability scores despite high taste scores, indicating that some bitter peptides were produced at lower levels of enzymatic hydrolysis. As a result, the composite gel M-2 (DH = 0.5%) showed the best texture performance and overall acceptability, while also helping to improve the color of the gel.

## 3. Conclusions

The protein structure of papain-modified SPI was more open and unfolded. The springiness, hardness, cohesiveness, and chewiness of the modified composite gels were higher than those of the control group, and the composite gels were directly crosslinked with TG (without papain modification of SPI). This is probably because the unfolding of the protein structure exposed the hydrophobic groups inside the protein molecule, thus promoting the interaction of SPI with SCP. The addition of modified SPI increased the WHC of composite gels. FTIR analysis showed that papain treatment resulted in the conversion of the β-turns into α-helices and random coils of SPI into β-sheets. These formed a denser gel network structure using covalent and hydrophobic interactions. However, excessive enzymatic hydrolysis resulted in smaller peptides that were not able to crosslink using TG. The molecular force results showed that hydrogen bonding, disulfide bonding, and hydrophobic association are important molecular forces for forming the SPI/SCP gels. The addition of the modified SPI increases the hydrogen bonds and the disulfide bonds. SEM analysis showed that the modified composite gels were more uniform, smoother, and had smaller pores and denser structures than the control group and the unmodified composite gels. In particular, the effect was most significant when the SPI DH was 0.5% (i.e., gel sample M-2). This indicated that the addition of modified SPI was conducive to the formation of SCP gel. The sensory evaluation of the gel samples was consistent with the results of the instrumental analysis. The quantitative results of sensory evaluation further indicated that the modified composite gel was likely to be highly acceptable to consumers. These results indicated that partial enzymatic hydrolysis allowed the successful use of TG, thus forming SPI/SCP composite gels with improved taste and gel properties, promoting the quality of SCP gels. In the future, the processability, thermal variability, thixotropic behavior, etc., need to be further investigated, as well as the biosafety of gel products (e.g., by animal testing, microbial detection, etc.), aroma substances and mechanism, and even more detailed structural analysis (X-ray diffraction and subunit composition) need to be clarified. Finally, all of these studies will constitute a complete, detailed, and comprehensive discussion and analysis to provide a theoretical basis for effectively improving the nutritional and functional properties of food.

## 4. Materials and Methods

### 4.1. Materials

Materials: SPI (protein content, 98%) was provided by the Xu Yang Experimental Supplies Co., Ltd. (Harbin, China). Live silver carp (*n* = 4) with an average weight of 1000 ± 100 g were sourced from a local aquaculture farm (Wuchang Shi, Harbin, China). Within 1 h, they were transported to the laboratory in closed expanded polystyrene boxes containing flaked ice (1:1). The live fish were killed using a welfare-friendly slaughter method. The silver carp were stunned with a blow to the head at room temperature (~20 °C), the head, internal organs, bones, and tail were removed with a knife, and the rest of the fish was washed with tap water. The knife was also used to fillet the fish, i.e., to remove the skin, backbone, and dark muscle to obtain the white muscle for subsequent SCP extraction. Glutamine transaminase (TG; 100 units (U)/mg), papain (800 U/mg) from papaya, and bovine serum albumin (BSA) were purchased from Solarbio & Technology Co., Ltd. (Beijing, China). Other reagents were obtained from Xu Yang Experimental Supplies Co., Ltd. and were of at least analytical grade.

### 4.2. Preparation of Papain-Modified SPI

SPI powder (15.0 g) was mixed with 150 mL of distilled water at room temperature (22 ± 1 °C) for 2 h with stirring using a magnetic bar. Then, the pH was adjusted to pH 7.0 using 0.1 mol/L NaOH, heated to 50 °C, and papain was added (E/S = 0.05% (*w*/*w*)) to start the hydrolysis process. The NaOH was used to maintain a constant pH (variation ± 0.1) during hydrolysis. The DH was determined using a pH-stat method, as described by Adler-Nissen [48]. Protein hydrolysis is accompanied by the release or absorption of protons depending on the pH. The DH can be calculated from the amount of base or acid added to maintain the pH of the system. The DH of 0.1, 0.5, 1.0, 1.5, and 2.0% were designated as DH0.1, DH0.5, DH1.0, DH1.5, and DH2.0, respectively. After hydrolysis, the samples were inactivated at 90 °C for 10 min and cooled to room temperature. The SPI dispersions were lyophilized (frozen at −30 °C, FD5-3, SIM Corp., Los Angeles, CA, USA) and stored in a desiccator for a maximum of one month.

The DH was calculated as follows:

$$DH=\frac{{B\times N}_{b}}{\alpha \times h_{tot}\times M_{p}}$$

where N_b_ is the equivalent concentration of NaOH, B is the volume of NaOH added (mL), h_tot_ is the total number of peptide bonds/g protein (taken as 7.78), α is the dissociation of α-NH_3_^+^, and M_p_ is the total protein (assuming 98% purity).

### 4.3. Preparation of Silver Carp Protein

SCP was extracted from the white muscle according to Mehdi et al. [49], with slight modifications. In brief, the silver carp muscle (200.00 g) was minced with 4 vol of sodium phosphate buffer (50 mM, pH 7.5) for 2 min using a high-speed plant crushing machine (JJ-2, Danrui Experimental Instrument Equipment Co., Ltd., Changzhou, China). It was then homogenized, using an IKA Homogenizer (T18, Jiading Analytical Instrument Co., Ltd., Qingdao, China), 4 times for 30 s each time (9000 rpm) and rested for 10 s, followed by centrifugation (8500× *g* for 15 min, 4 °C) (GL21M, Xiangyi Centrifuge Instrument Co., Ltd., Changsha, China) to remove water-soluble protein. This sequence was repeated 3 times. The water-soluble protein was then mixed with 4 vol (*w*/*v*) of 0.1 M NaCl and filtered through four layers of gauze (Xu Yang Experimental Supplies Co., Ltd.) before centrifugation to remove connective tissue. This sequence was repeated a second time. The protein concentration of the SCP was measured using the Biuret method [50], with bovine serum albumin as the standard protein assuming 100% purity. The SCP was stored at 4 °C and utilized within 48 h.

### 4.4. Preparation of Cold-Induced Gels

SCP suspensions (final protein concentration of 40 mg/mL) were prepared with 50 mM PIPES buffer containing 0.6 M NaCl (pH 6.25). N-SPI or papain-modified SPI was added to the suspensions to obtain final SPI concentrations of 0.75% (*w*/*v*) after mixing. These mixed protein solutions in 50 mL centrifuge tubes were centrifuged (800× *g*, 1 min, and 4 °C) to remove bubbles. Then, TG (20 U/g) was added, the mixture was transferred to a cylindrical gel bottle (25 × 40 mm), and the gel bottles were put into a constant temperature water bath (50 °C, 2 h), followed by a 90 ℃ water bath for 10 min to stop the reaction. After the samples were cooled, they were stored at 4 °C overnight to form a cold-induced gel. The gels formed with SCP alone were the control, the gels formed by the combination of SCP and N-SPI were designated M-0, and the gels of SCP with papain-modified SPI with DHs of 0.1, 0.5, 1.0, 1.5, and 2.0% were designated as M-1, M-2, M-3, M-4, and M-5, respectively. These samples were equilibrated at room temperature for 30 min before the gel properties were measured.

### 4.5. FTIR (Fourier-Transform Infrared Spectroscopy)

The freeze-dried samples were crushed into a uniform powder using a mortar. Each sample (3 mg) and 100 mg of KBr powder were mixed and then pelleted using a tablet press machine (FW-4, Tianguang Optical Instrument Co., Ltd., Tianjin, China). The samples were scanned 32 times from 400–4000 cm^−1^ at a resolution of 4 cm^−1^ (Nicolet iS10 FTIR spectrometer, Thermo Fisher Scientific, Waltham, MA, USA). The amide I band was scanned from 1600 to 1700 cm^−1^. The second derivative spectrum was obtained using Peak Fit v4.12 derivative function software (Origin Lab Corp., Waltham, MA, USA). The secondary structure components of the sample were quantitatively analyzed using a method of “peak fitting”, assuming that there were only four possible structures, i.e., α-helix (1650–1660 cm^−1^), β-sheet (1618–1640 and 1670–1690 cm^−1^), β-turn (1660–1700 cm^−1^), and random coil (near 1645 cm^−1^) [23,34].

### 4.6. TPA (Texture Profile Analysis)

The composite gel TPA test followed the method of Mehdi et al. [49] with some modifications. After the gel sample was equilibrated in the gel bottles at room temperature, it was cut at both ends with a knife into a cylinder with a diameter of 25 mm and a length of 30 mm. The samples were tested using a TA-XT2 texture analyzer (Stable Micro Systems Ltd., Godalming, UK) using a P/36R cylindrical plate (36 mm diameter) using the following parameters: pretest speed of 2.0 mm/s; test speed of 1.0 mm/s; posttest speed of 2.0 mm/s; and trigger force of 5 g. The texture parameters (hardness, springiness, chewiness, and cohesiveness [29] were obtained after a 50% deformation. The analysis was repeated three times with a new gel each time for each composite gel sample, and the software with the instrument was used to calculate the texture parameters.

### 4.7. Gel Whiteness

The whiteness of the composite gels was measured using a color meter (ZE-6000, Nippon Den-Shoku, Inc., Tokyo, Japan). The instrument was calibrated using a standard white reflector board. The samples were placed on the instrument and covered with the zero-correction cup. The whiteness was calculated from the L (brightness), a (red to green), and b (yellow to blue) values. The formula was as follows [33]:

$$Whiteness=100-\sqrt{{\left(100-L\right)}^{2}+a^{2}+b^{2}}$$

### 4.8. Gel WHC (Water-Holding Capacity)

The gel samples (5 g) were placed in polypropylene conical tubes (50 mL) and weighed (W_1_). Then, the gel samples were centrifuged (3000× *g*, 4 °C, 15 min). The water was removed and the centrifuge tube with the sample was weighed after centrifugation (W_2_). The WHC (%) was defined as the ratio of remaining water after centrifugation to the original water in the gel, multiplied by 100. The total weight of water in the gel sample was measured using oven heating (W_0_) at 105 °C for 2 h. The weight of the water removed from the gel sample was W_r_ (W_r_ = W_1_ − W_2_). WHC was determined using the equation [51]:

$$WHC=\frac{{W_{0}-W}_{r}}{W_{0}}$$

### 4.9. Gel Sample Intermolecular Force Measurements

Non-covalent and covalent interactions of the composite gels were studied [52]. Four denaturing solutions were used: 0.6 M NaCl (S1) to detect ionic bonds, 0.6 M NaCl + 1.5 M urea (S2) to detect hydrogen bonds, 0.6 M NaCl + 8 M urea (S3) to detect hydrophobic interactions, and 0.6 M NaCl + 8 M urea + 0.5 M β-ME (S4) to detect disulfide bonds. The composite gels (0.5 g) were mixed with 10 mL of each denaturing agent (pH = 7.0) mentioned above to determine their solubility and were then homogenized (IKA-Werke GmbH & Co., Staufen, Germany) at a speed setting of 8 (~18,000 rpm) for 2 min. The composite gel samples were then centrifuged (TGL-16M) at 12,000× *g* for 30 min at room temperature. Using BSA as the standard assuming 100% purity, the protein concentration in the supernatant was determined using the Coomassie brilliant blue method [52], and the results were reported as BSA equivalents (E). The solubility of S1 represents the contribution of ionic bonds, and the differences in gel solubility between solvents S2 and S1 mainly represented hydrogen bonds. The differences in gel solubility between solvents S3 and S2 mainly represented hydrophobic interactions, and the differences in gel solubility between solvents S4 and S3 mainly represented disulfide bonds. Each S4 fraction was dialyzed against solution S3 for 24 h at room temperature using a 3000 nominal molecular weight cut-off tube (Thermo Fisher Scientific) to avoid interference from β-ME.

### 4.10. Gel Sample SEM (Scanning Electron Microscopy) Measurements

Following Xu et al.’s method, SEM (SU6600, Hitachi High Technologies Corp., Tokyo, Japan) was used to analyze the composite gel microstructures [53]. The composite gels were cut into small strips (2 × 5 mm^2^) using a sharp razor blade. After a series of rinses, the composite gels were freeze-dried (24 h) using a critical point dryer (CPD03, Balzers, Alzenau, Bavaria, Germany), sputtered and gold plated using an SCD 050 Sputter Coater (Balzers), and then observed at an acceleration voltage of 5.0 kV.

### 4.11. Sensory Evaluation

The composite gel products were evaluated by 10 men and 10 women who regularly tested the product. To improve objectivity, the sensorial panel was trained and became familiar with the scoring system [54]. The composite gels were cut into 2 × 2 × 1 cm pieces with a knife at room temperature before each session, and evaluated on a white paper plate with a three-digit random code. To eliminate any order effects, the order of the composite gel samples was randomized and only one gel sample was given at a time. The panelists were given a glass of water (Xu Yang Experimental Supplies Co., Ltd.) as a palate cleanser between samples.

### 4.12. Statistical Analysis

All experiments were repeated 3 times, and the data are shown as the mean values ± standard deviation (SD). The data were analyzed for significant differences using the Statistic Package for the Social Sciences (SPSS) software (Version 20, SPSS Inc., Chicago, IL, USA), and the figures were plotted using Origin Pro 2016 64-bit software (Origin Lab Corp., Northampton, MA, USA). *p* < 0.05 was considered significant.

## Figures and Tables

**Table 1:** Results of secondary structure contents in samples.

Gel Sample	α-Helix (%)	β-Sheet (%)	β-Turns (%)	Random Coil (%)
Control	26.1 ± 0.2 ^e^	31 ± 1 ^c^	25.4 ± 0.4 ^a^	18 ± 1 ^a^
M-0	27.1 ± 0.3 ^bc^	31 ± 1 ^c^	24.3 ± 0.2 ^b^	17.6 ± 0.3 ^abc^
M-1	26.8 ± 0.4 ^bcd^	33 ± 1 ^bc^	24.1 ± 0.1 ^b^	17.8 ± 0.2 ^ab^
M-2	28.4 ± 0.2 ^a^	36 ± 2 ^a^	22.0 ± 0.1 ^d^	15.2 ± 0.1 ^e^
M-3	27.4 ± 0.1 ^b^	35 ± 1 ^ab^	22.2 ± 0.3 ^d^	16 ± 1 ^de^
M-4	26.4 ± 0.3 ^cd^	34 ± 1 ^ab^	23.4 ± 0.1 ^c^	16.9 ± 0.2 ^bcd^
M-5	27 ± 1 ^bcd^	32 ± 2 ^bc^	24.4 ± 0.4 ^b^	16.6 ± 0.4 ^cd^

Note: Control gels: the gel formed using SCP alone. M-0: the gel formed using the combination of SCP and N-SPI. Gel samples M-1, M-2, M-3, M-4, and M-5: the gels of SCP with papain-modified SPI at different degrees of hydrolysis of 0.1, 0.5, 1.0, 1.5, and 2.0%. Significant differences *p* < 0.05 are indicated by different lowercase letters in the same column.

**Table 2:** Evaluation properties of the sample.

Sensory Attribute	Definition and Description	Rating Scale
Elasticity	Whether the gel sample has good elasticity; press with your finger and observe whether it breaks.	1–3 = If you press the sample with your finger, it breaks, which represents an inelastic state. 4–6 = When the sample is pressed with the fingers, it is less fractured and shows a slightly elastic state. 7–10 = The sample does not break when pressed with fingers; it shows good elasticity.
Mouthfeel	Gel samples are refreshing, smooth, and delicate, or not, when coming into contact with your tongue.	1–3 = Unpleasant, nonsmooth, and rough. 4–6 = Slightly refreshing and smooth, and slightly delicate. 7–10 = Refreshing, smooth, and delicate.
Color	Whether or not the gel sample has the normal color (light white or light yellow), and whether or not the color of the sample is uniform.	1–3 = The sample has an abnormal color. 4–6 = The sample has a normal color but the color is uneven. 7–10 = The sample has a normal, uniform color.
Taste	Whether the gel sample is delicious and has enough flavor.	1–3 = Not delicious, with an unpleasant smell. 4–6 = Delicious, slight flavor. 7–10 = Delicious, enough flavor.
Apparent state	The gel sample has a uniform cut surface, with pores or not.	1–3 = The surface of the cut is fluffy and uneven. 4–6 = Slightly uniform cutting surface with a few large pores. 7–10 = Smooth cut surface, no large pores.
Acceptability	Whether or not the gel sample has a bitter taste, and what is the overall taste and acceptability?	1–3 = The gel sample has a bitter taste and poor acceptability overall. 4–6 = The gel sample has a slight bitterness, and overall acceptability can be considered. 7–10 = The gel sample has no bitterness, and overall acceptability is good.

**Table 3:** The result of the sample evaluation scores.

Gel Sample	Elasticity	Mouthfeel	Color	Taste	Apparent State	Acceptability
Control	3.4 ± 1.1 ^e^	2.1 ± 0.3 ^c^	6.1 ± 1.2 ^bc^	4.1 ± 0.4 ^c^	3.3 ± 0.2 ^d^	5.3 ± 0.4 ^e^
M-0	3.0 ± 1.0 ^e^	1.7 ± 0.2 ^c^	5.3 ± 0.4 ^c^	3.4 ± 0.4 ^c^	3.1 ± 0.4 ^d^	4.6 ± 0.4 ^e^
M-1	5.2 ± 1.2 ^cd^	6.3 ± 1.4 ^ab^	6.4 ± 0.4 ^abc^	6.1 ± 0.1 ^b^	7.0 ± 1.0 ^bc^	7.3 ± 0.2 ^bc^
M-2	8.3 ± 1.3 ^a^	7.6 ± 1.2 ^a^	7.6 ± 0.2 ^a^	8.4 ± 0.2 ^a^	8.7 ± 0.2 ^a^	8.8 ± 1.0 ^a^
M-3	7.1 ± 0.3 ^ab^	7.0 ± 1.1 ^a^	7.0 ± 1.0 ^ab^	7.2 ± 1.1 ^b^	8.1 ± 0.4 ^ab^	8.1 ± 0.2 ^ab^
M-4	6.2 ± 0.4 ^bc^	6.1 ± 0.4 ^ab^	6.7 ± 0.3 ^ab^	7.0 ± 1.2 ^b^	7.3 ± 1.3 ^bc^	6.6 ± 0.5 ^cd^
M-5	4.4 ± 0.4 ^de^	5.3 ± 0.1 ^b^	6.4 ± 0.2 ^abc^	6.2 ± 0.3 ^b^	6.4 ± 0.4 ^c^	5.5 ± 1.1 ^de^

Note: Control gels: the gel formed using SCP alone. M-0: the gel formed using a combination of SCP and N-SPI. Gel samples M-1, M-2, M-3, M-4, and M-5: the gels of SCP with papain-modified SPI at different degrees of hydrolysis of 0.1, 0.5, 1.0, 1.5, and 2.0%. Different lowercase letters in the same column indicate significant differences; *p* < 0.05.

## Data Availability

The data presented in this study are available in [Gel properties and structural characteristics of composite gels of soy protein isolate and silver carp protein].
